# A blended model of interior design studio practice due to COVID-19

**DOI:** 10.12688/f1000research.128478.2

**Published:** 2023-06-20

**Authors:** Imad Assali

**Affiliations:** 1 Interior Design Department, Ahila University, Manama, Bahrain

**Keywords:** Virtual Design Studio (VDS), COVID-19, E-learning, Blended learning, Virtual learning environments (VLSs), Moodle, MS Teams, Face-to-Face learning

## Abstract

**Background: **The coronavirus disease 2019 (COVID-19) pandemic upended the educational system around the globe. During this challenging period, universities and colleges looked for other effective alternative methods of learning, such as Virtual Learning Environments (VL). Besides, Ahlia University has implemented E-learning in response to COVID-19. There needs to be more attention given to the challenges associated with technology adoption facing interior design and architecture programs, where over 60% of courses are practical, especially design studios, which form the core of the curriculum. According to a review of the relevant literature, there needs to be more research on blended learning in interior design and architecture. In order to enhance the teaching and learning of interior design and architecture, further research is required to combine cutting-edge techniques and technology. The aim of this study was to review the classroom materials for Ahlia University's interior design studio.

**Methods: **After completing the INTD 212, INTD 216, INTD 311, and INTD 404 studios in mid-March 2022, a short Qualtrics poll was done to assess the difficulties of e-learning and offer potential consequences.

**Results:** Though students were conveniently attending courses online, there was not much discussion and interaction like in the face-to-face model. Blended teaching in design studio courses offered many benefits. The results showed that blended design studios achieved pedagogical results as students developed their knowledge.

**Conclusions: **Based on the findings, this research concludes that teaching and learning should be shifted from face-to-face and online learning to the best practice of a blended format.

## Introduction

*“A flexible learning approach aims to provide learners with excellent choice, convenience, and personalization to suit their needs. With flexible learning, learners can choose where, when, and how they learn through a variety of technologies that support teaching and learning.* *p.02”.* ^1^

The environment for interior design education is developing and reaching new heights due to cutting-edge technologies, innovative teaching strategies, and raised standards for design education.

However, worldwide interior design education needs to rapidly prepare for extreme situations, given the ongoing coronavirus disease 2019 (COVID-19) pandemic. However, to finish the academic year without squandering the current semester, colleges worldwide are switching to online and e-learning methods. Within a week of the first COVID-10 lockdown, online classes at Ahlia University started. However, neither the teachers nor the students anticipated how it would affect every course, academic or practical. ^2^^,^^3^

All educational institutions worldwide have shut down as the world fights COVID-19, which impacts learning. It is essential to offer learners a face to face learning method. ^4^^,^^5^ However, this conclusion highlights the significance of Virtual Learning Environments (VLSs) as a replacement for the conventional pedagogical strategies of lectures, tutorials, and exam to guarantee the effectiveness of education. The rise of information communication technology and the internet-savvy youth, which increased learning options during the COVID-19 epidemic, are prerequisites for these new learning techniques. ^6^^,^^7^ It emphasizes the necessity for faculty members knowledgeable about and skilled in online pedagogies and emphasizes student-centered practices more. ^1^ Like many other campuses, Ahlia University shut down its physical structure in reaction to COVID-19 to stop the virus’s spread. It set up various Microsoft Teams-based virtual professional development sessions for its faculty members who were taking their first e-learning courses to give them the assistance they needed to transition to virtual learning and get students ready for this change in environment. Students could access resources and submit their assignments after faculty members presented live lectures using the Microsoft Teams (MS Teams) platform, post-learning activities, and related resources on Moodle. Along with the traditional design studio (face-to-face learning), the mainstay of interior and architectural design education has been dealing with real-world problem-solving and developing students’ conceptual grasp of design since the 19th century. ^8^ Today’s design studio education is experiencing unheard-of difficulties due to new technology and COVID-19 constraints. It is necessary to incorporate technology tools, create online instruction, and evaluate students’ levels of understanding through synchronous and asynchronous interactions to design blended learning that uses face-to-face and virtual learning in a hurry. This will help to create a productive learning environment. ^9^^–^^11^ As cited by Brown *et al.* ^10^ from Hulon [Ref. 8: p.6], the studio is “ *a virtual world representing the real world of practice, but relatively free of its pressures and risks*”. Consequently, design studios prepare students for professional practice. Since the middle of the 1990s, the Virtual Design Studio (VDS) has grown significantly, urging attention to the potential of digital technologies and their influence on instructional strategies. ^12^^–^^14^ In order to better understand the preferences of the students in Ahlia University’s interior design program for blended, online, and face-to-face learning, this research examines the engagement of blended learning, faculty members’ roles, and students’ experiences in blended learning in interior and architecture design education. Investigate how blended learning can be used effectively in the interior design curriculum.

## Literature review

The design studio is the guiding principle of interior design education and other design disciplines. It serves as a model for educational innovation. It is devoted to teaching students how to construct products that develop their technical and social abilities by combining knowledge from the classroom with real-world experience. ^7^^,^^15^^,^^16^ The design studios have provided the development of students’ creativity, imagination, professional expertise, and post-graduate practical experience with the utmost relevance and legitimacy. ^16^^,^^17^ At all design education levels, a design studio entails one-on-one interaction between students and the studio educator, group discussions, and teamwork in creating design ideas that are ultimately presented to the studio educator and other invited experts’ audiences. ^18^ As a result of the coronavirus epidemic, design studios’ pedagogy has changed from face-to-face classes to online classrooms, posing a new challenge for students and faculty members who must provide the highest education as specified by the Council of Interior Design Accreditation. New technologies, social networking sites, and the emergence of artificial intelligence have revolutionized teaching and learning methods and facilitated distance learning that allows student autonomy through online learning models. The current and ongoing coronavirus situation is also a result of these changes. ^18^ Despite the pandemic affecting all design schools in late 2019, e-learning has always been part of design studios and has proliferated in all design schools, particularly virtual design studios (VDSs). Faculty and students use MOODLE, Microsoft Teams, MOODLE, and other platforms to present online, distribute class materials, and submit final work.

### Designing blended learning courses

A hybrid or mixed-mode course that combines traditional in-person instruction with online learning is known as blended learning. In addition, blended learning (also known as hybrid or mixed-mode courses) is a competency-based paradigm that combines traditional face-to-face instruction with online learning.

According to Schnabel *et al*., ^11^ blended learning refers to a hybrid course that substitutes technology-supported activities for a portion of the standard course’s time. According to Troha, ^19^ blended learning is the term for online and in-person activities. They continued by defining course objectives before course activities, such as assignments and assessments, as they determined the delivery method between in-class and online activities. Blended learning, they said, refers to the combination of on-campus class meetings and online activities. Here, the question of how much in-person instruction must be replaced with online coursework arises. McGee and Reis ^18^ estimates that 30% and 79% of blended learning lessons are provided online. It is challenging for studio-based learning to have a large student enrollment since a traditional studio environment has a small student-to-studio educator ratio where students and their tutors interact in a creative and reflective process. Therefore, blended learning can offer a comprehensive enrollment studio course to address this difficulty. It also overcomes the limitation of physical classroom space in some universities and courses that may not lend themselves to online delivery, such as labs. ^11^ According to McGee and Reis, ^18^ modern technology that eliminates physical barriers between students from different cultures encourages collaboration. It allows collaboration on design projects with other design schools worldwide. Besides the physical distance, tutorials, and desk crits, studio instructors’ roles in both VDSs and traditional design are similar. However, despite employing various mediums, students must present design solutions. As cited in the BlendKit Course, ^5^ “ *Students in online conditions performed modestly better, on average than those learning the same material through traditional face-to-face instruction*” and, “ *Instruction combining online, and face-to-face elements had a larger advantage relative to purely face-to-face instruction than did purely online instruction.* The University of Central Florida (UCF) has been using blended learning for over 17 years and has offered 10,941 blended courses since the end of the 2015-2016 academic year. Despite these benefits, the virtual studio procedure is more complex than it seems because it is almost certainly more complicated and time-consuming than face-to-face studios. According to Schön, ^16^ students construct ideas in a virtual design studio that respond to the issue in the design brief and offer solutions, typically by utilizing different software like AutoCAD to make digital drafts, which takes more time than creating hand-drawn sketches. Students must therefore have extensive training in interior and architectural design and comprehensive education in ICT. ^12^ In order to help their students, faculty staff must also be knowledgeable in ICT and online instruction. ^20^ According to Schön, ^16^ social media and digital tools help people develop their talents, making it simple to implement virtual design studios (VDS) into design studio education. Students in interior design and architecture schools employed software tools like AutoCAD, 3DMax, Building Information Modeling, and Adobe Photoshop in their design projects at various design studio levels. ^12^

## Problem statement

The idea of virtual and online design studio classrooms for interior design raises several issues, focusing on the communicative and practical components of learning. The COVID-19 global lockdown has significantly impacted the practical, communicative, and team-building scenarios that are part of interior design studio classes. Universities worldwide have a sense of losing credibility in the design studio when they fall short of the ideal quality of global design education. Maher and Simoff ^14^ emphasized that the studio needs to be an appropriate size to ensure a direct connection between students and their studio educators and each other. It is therefore vital to understand and adapt to the extensive extent to which design studios will change in the future.

## Objectives

This study aims to understand the crucial changes and measures that virtual design studios will have to adapt to due to the current COVID-19 pandemic in interior design and architecture education. By assessing the results of the analysis presented to the faculty and students, this study also aims to understand their perspectives on online and e-learning education. Thus, the main summary of the research objectives is:

• Integrating physical and online education in interior design courses in order to determine the success rate.
• Developing comprehensive solutions to make online and e-learning education a global norm.
• To meet the education standards set by the Council of the Interior Design Accreditation (CIDA) during this challenging time of virtual and e-learning methods being the only feasible and safe options available.
• Emphasizing and demonstrating the importance of how virtual design studios (VDSs) can enhance internships and post-graduate jobs for students.

For academic research to be credible and accurate, bias must be avoided. In this study, efforts were made to address potential sources of bias, including discussing the results with the responders.

## Methods

### Study design

Based on the abovementioned research objectives, this study’s primary goal is to evaluate the effectiveness and standard of blended learning in design studio procedures as a result of COVID-19. Thus, this pilot study used a blended learning environment that combines a face-to-face studio environment with an online studio environment for design studio courses. The learning environment includes INTD 212, INTD 216, INTD 311, and INTD 404 of interior design students from different levels, faculty members, and interior and architecture professionals, along with appropriate journals and literature assessments over five months. It was conducted over ten weeks in the spring semester of 2020.

### Sample

Participants were selected in all design courses (probability sampling).

### Variables

The goal was to compile and watch the details of participants’ behaviors in the blended learning setting. The author of this work did the observation by visiting some classes at Ahlia University face-to-face and through MS TEAMS. Students’ confidence level, access to the Internet during online education, convenience in using MOODLE, Microsoft Teams, computer-aided software or developing projects, students’ listening abilities, students’ progress on projects, students’ response to the online environment, and increased opportunities for interaction and collaboration with peers are the factors considered for this study. ^24^^,^^25^ Further, the factors observed in this study were the impact of online education allowing students to communicate with other students better than a conventional face-to-face teaching model. It is overall monitoring between the instructor and the students through discussion, feedback, and filling of the survey questions.

Participants were selected from all design courses: INTD 212, INTD 216, INTD 311, and INTD 404 (probability sampling). Students were positive and satisfied with taking part in the study, both on the implementation of blended learning. Students were also permitted to reopen the lesson without any pressure. A total of Three hundred and fifty-seven undergraduate students (283 females and 74 men) enrolled in interior design and architecture courses at six institutions in Bahrain responded to the survey.

In addition, in-depth interviews with eighteen (18) teaching staff (11 women and 7 men) about the students’ awareness and cognition of the difference between online and blended design studio courses were carried out. A survey was also conducted which examined students’ software literacy in a design project and their preparedness for online learning. ***Moodle and LMS*** make it easy for students to look back on earlier materials and to move through coursework at their own pace. It also examines studio lecturers and students’ satisfaction with Moodle and MS Teams in communication, feedback, and project progress using Likert scales.

### Ethical considerations

After preparing and refereeing the tools for data collection, a written request was made to the Deanship of Graduate Studies and Research (DGSR), Scientific and Ethical Committee, Ahila University, who approved the study on 22 ^nd^ March 2020. Students, the core of the study, were given the purpose of the research and asked to participate in the study. A form containing details of the project, such as the objective and duration of the study, was given to the students from the design courses: INTD 212, INTD 216, INTD 311, and INTD 404. A total of Three hundred and fifty-seven undergraduate students agreed to participate in this study. After the participants filled a written statement of willingness to participate in the study. We held a zoom meeting with students who agreed to participate in the study. We explained the objectives of the survey and informed them that they were requested to provide feedback on blended learning. Similarly, a detailed explanation of the project was provided to the 18 staff members willing to participate in the study, and written consent was obtained from them. The researcher told them that all their information would remain anonymous and would not be used for other purposes.

### Materials

Design work was done on MOODLE and Microsoft Teams throughout the design studio courses, using both synchronous and asynchronous interaction platforms, such as lectures, discussions, announcements, presentations, project briefs, research studies, and sharing images, drawings, and evaluation criteria.

The questionnaire via Google Online Surveys includes closed and open questions for all students in these four design studio classes. ^25^ It uses qualitative and quantitative approaches to examine student opinions on the methodology and tools employed. Closed questions were used to gather information on demographics and e-learning preferences. ^8^ In order to understand more about how students, interact with the various e-learning platforms, open-ended questions on learning and teaching in higher education were posed to the participants.

The study considers adding some questions that was earlier presented by Hulon *et al.* ^8^ This is our initial attempt to focus on studying the face-to-face and online learning and blended format. Therefore, prior to implementation of the pilot study of our survey, a panel of experts from our university were provided with questions and they were asked to provide feedback on how well each question measure the construct in question. Since this is our preliminary pilot work, we have developed the questions and the experts reviewed it. In analyzing the data, we ensured that the questions all reliably measure the same latent variable. We employed SPSS’s Version 27.0.1.0 reliability command to test for internal consistency using Cronbach’s alpha. Our interpretation of the output was based on George and Mallery’s (2003) rule.

### Data analysis

Of the Three hundred and fifty-seven undergraduate students who agreed to participate in the study, it has continued for over ten weeks of the project duration, and all the students who participated filled out the survey questions. None of the patients withdrew or dropped out of the study. Interview details from 18 staff members and data from their responses were collected for analysis. The student’s t-test was used to analyze the data. Student’s t-test was used to compare the effectiveness of online, face-to-face, and blended teaching. When performing a student’s t-test for online and face-to-face teaching comparisons, the significance level was set at 0.05. This means that the results of the t-test must be statistically significant to a level of 95% confidence. SPSS Version 27.0.1.0 Statistical Software Package for analyzing the data.

Enhancing statistical analysis methods through advanced analytics is essential to provide designers with the data-driven insights they need to create more valuable and customer-centric interior design solutions. It is a powerful tool that provides the ability to easily perform intricate analyses. Moreover, it includes a comprehensive range of statistical procedures that are useful for analyzing complex datasets. With the onset of the COVID-19 pandemic, interior design studio practices have been significantly impacted. The adoption of a blended model can help in mitigating the effects by enabling online collaboration. Using SPSS version 27.0.1.0 to analyzing data from the blended model can enable practitioners to derive meaningful insights from the collected data.

### Integration of Moodle and Microsoft Teams

The use of Moodle and Microsoft Teams for supporting learning and teaching that provide chat, virtual meetings, shared files, and the ability to organize live sessions synchronously or asynchronously is advised in response to COVID-19 restrictions forbade face-to-face instruction when the lockdown started in March 2020. The Ahlia University IT staff quickly delivered training and thorough instructions on using the new Microsoft Teams. As a result, the instructors in the interior design program at Ahlia University (AU) used Microsoft Stream and Moodle as communication tools for both theoretical and design studio courses like INTD 212, INTD 216, INTD 311, and INTD 404 in the spring semester (2020). They discovered its effectiveness in the delivery of the courses.

## Results

Online teaching has become increasingly popular in higher education. Online teaching is becoming increasingly popular in higher education and can be a challenge for staff that have not previously taught in this format. Our staff reported when conducting interviews that teaching a design studio course online requires demonstrating both technical and creative skills. While there are many positive aspects of online learning, such as increased accessibility, they encountered difficulty in transferring the design studio course from an in-person setting to an online one. Staff reported that they had struggled to create an engaging online environment for students and to ensure that the course was just as enriching as if it were taught in person. Additionally, the lack of face-to-face interaction in an online setting makes it difficult to build relationships with students. They reported that they faced difficulty in understanding how well each student was performing and in providing the necessary feedback. Another concern is that Staff they had some issues when technical difficulties arise during an online lesson which included on learning how to use the various software programs, troubleshooting any connectivity issues. All these elements were challenging for the staff to effectively teach an online design studio course.

Based on probability sampling, the students were selected from the following design courses: INTD 212, INTD 216, INTD 311, and INTD 404. A total of 357 students participated in this study. ^24^ The participants were informed of the study objectives. All the students who showed interest in participating in the study gave their feedback, and none of the students dropped out during the course of this study. As an initial step in validating the survey is to establish face validity. The experts related to our study read through the questionnaire completely and evaluated whether the questions effectively capture the objective. Our interpretation of the output was based on George and Mallery’s (2003) rule. Cronbach’s alpha coefficient increases as the number of variables increased, and the average inter-item correlations increased.

**Figure 1.**
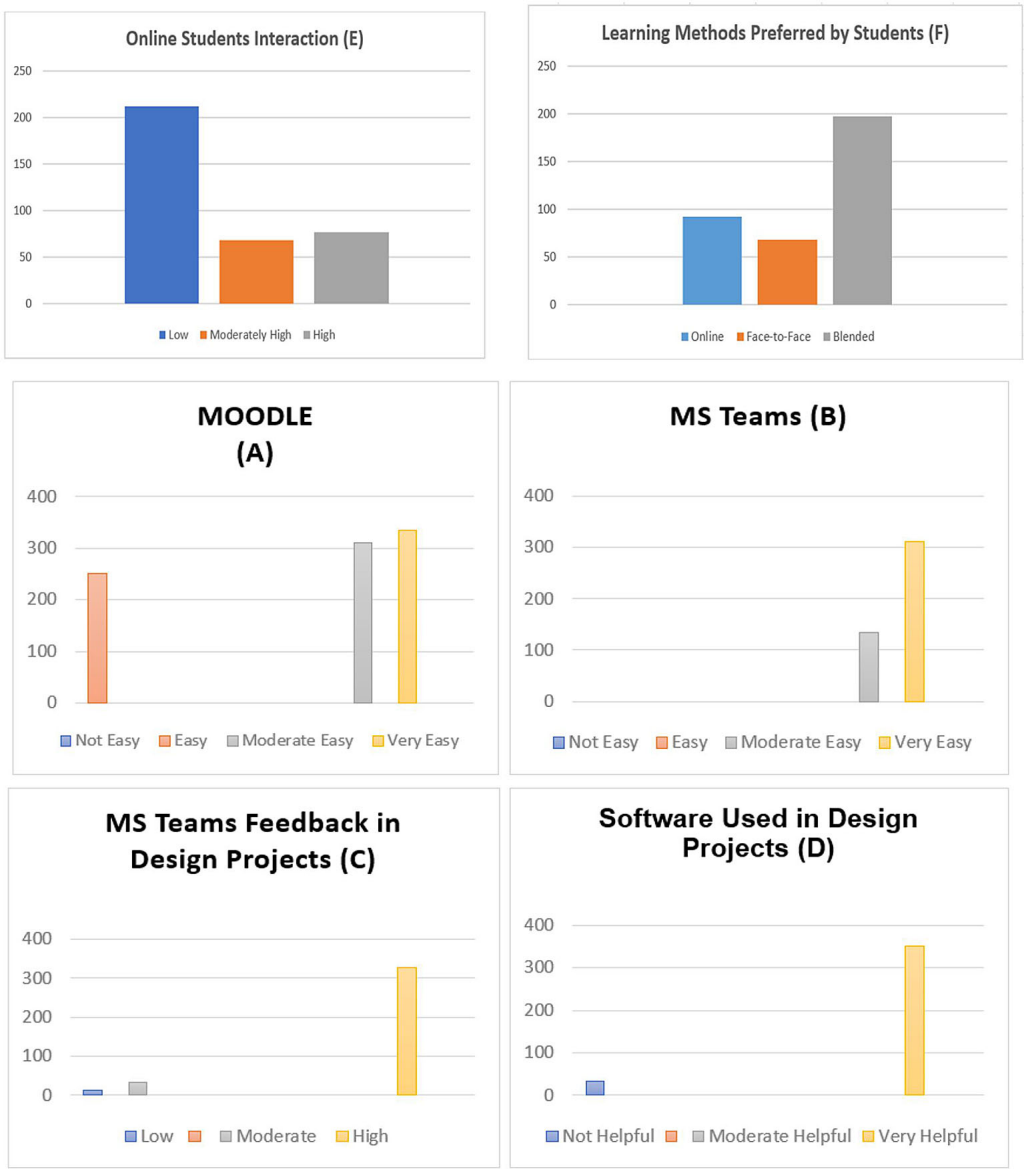
This figure illustrates the satisfaction levels of online platforms (A) and (B), feedback from MS Teams for design projects (C), software used for design projects (D), and online student interaction (E), learning methods preferred by students (F).

Using quantitative analysis for 357 students, 79.2% (283) were women, and 74 were men, from different design departments in Bahrain. The questionnaire included information about the students’ degree in digital literacy, showing that 98% of them had access to a computer at home for academic purposes. The fact that 73% of students used social media daily, 71 used it sometimes, 14 typically used it once per week, and 11 said they did not use it at all demonstrated their suitability for design studio E-learning education. E-learning environments were suitable for design education by 72% of the respondents. In contrast, blended learning environments—including instructor and technical support from the university—were deemed the most effective and suitable in 78.5% of the responses. The results demonstrated a significant impact on the quality of online design studios. Besides, more than 73.8% of the respondents affirm that they have become more proficient in computer software, which helped them in their design projects as online learning provides them with the flexibility of time to develop their skills. Overall, 82 of the respondents reported that face-to-face teaching is crucial in design education. On the contrary, first-year students in all the universities in Bahrain were less satisfied with online learning ( Figure 1A), especially in courses like graphic design and basic design. Also, findings about MS Teams and Moodle revealed that 94% of the respondents were satisfied with these platforms ( Figure 1B). They provide satisfactory results because they are easy to use and effective for online design studios for communication, discussion, and the exchange of design information, enabling studio educators to monitor student work ( Figure 1D).

According to the survey, students share their design creations online to receive feedback and criticism from studio educators. The findings showed that 96.7% of students choose not to post their design work on Moodle or MS Teams ( Figure 1E), keeping it instead for in-person meetings with their studio instructor or uploading it as soon as it is finished out of concern for intellectual theft. Furthermore, online learning saves students’ travel time to the campus in developing their technical skills for design projects. The survey results revealed that 11 participants said they had trouble downloading and uploading files and connecting to the internet. Smaller class sizes in design courses would be preferable, according to 32 of those surveyed, while 64 students noted e-learning and blended learning as more novel and interactive. In this way, the questionnaire results provided insightful information about how students had used Moodle and Microsoft Teams to study effectively in a hybrid environment ( Figure 1F). In addition, students believe that in-person instruction is a crucial component of design education.

Online learning could lead to a more flexible, student-centered, and creative educational process. The role of faculty members will be transformed from the traditional teacher-centric model to a student-centric model with the implementation of e-learning. Implementing online learning has increased globally recently, and some higher education institutes in developing countries are embracing this trend. This study aimed to investigate the perception, challenges, and predictors of online and face-to-face learning acceptance during the COVID-19 pandemic among university students. We found that most of our participants agreed that the online course offered greater flexibility in teaching times. In contrast, many participants reported that online learning could take time and lead to listening difficulties and experienced network and technological problems affecting learning progress.

During online education, most participants were satisfied with the effort involved in developing a project using computer-aided software. According to 72% of respondents, e-learning environments are suitable for design education. In contrast, first-year students were less satisfied with online learning, particularly in graphic design and basic design courses. Despite this, 96.7% of students chose not to post their design work on Moodle or MS Teams, instead choosing to present it in person with their studio instructors or upload it as soon as possible to avoid intellectual theft. According to the study, 89.1% of participants strongly agreed that instructors could teach at their own pace through an online course. Although they are geographically separated from the instructor, they feel connected to the course and the instructor.

By combining face-to-face and online learning, blended learning offers learners the benefit of both learning methods. Studying asynchronously enables participants to study at their own pace, but questions are generally posted on an online forum so instructors can answer them. As a result, interactions are delayed. During face-to-face teaching, instructors and students are in constant communication, allowing for instant discussions and clarifications of questions. Online learning has numerous benefits, but many learners still prefer face-to-face learning due to the discipline and familiarity it offers. Most study participants reported that online education prevented them from communicating effectively with other students. Most participants felt they could communicate with other students in a conventional face-to-face approach.

The outcomes of this study revealed that online learning is more flexible than traditional learning, and face-to-face learning is generally more practical for learners. The majority of our study participants preferred blended learning (both online and face-to-face) for developing knowledge and skills and better outcomes.

According to 86.7% of the students, blended learning gives them flexible time and lets them study when it is convenient. Additionally, it allows them to communicate with their studio educator for guidance and feedback more frequently than in a face-to-face setting, which enables them to advance their design project more quickly. As cited by some students, “ *it is such a help to know the next studio whether there is something to improve on, or just to ask simple questions that would not be answered until the next studio. It also allows you to express your ideas more in e-learning to make sure you fully get your point across. You can forget things easily in the studio*”.

A quick review of the teaching staff revealed that all of them had access to computers at home, used online learning environments like Moodle and Microsoft Teams for educational reasons, and took part in various virtual workshops and conferences utilizing the Zoom platform. The survey also showed that 93% of respondents used social media sites like Facebook, Twitter, and Instagram, which Ahlia University offers, to communicate. During the pandemic epidemic, all interior design and architecture professionals’ work was moved to the virtual world by employing computer-mediated collaboration platforms to discuss design projects with their clients and contractors. Therefore, interior design and architecture programs use the same approach to prepare their graduates for the market needs.

## Discussion

In this study, both the students and staff reported that both online teaching and face-to-face teaching have advantages and limitations when used in a design studio course. Online teaching can provide more flexibility and convenience as students can access resources, such as video tutorials and lectures, from wherever they are, and can complete their work at their own pace. However, the lack of direct contact with instructors and peers can be a major limitation of online teaching in a design studio course, as it can be difficult to replicate the traditional in-person learning experience. On the other hand, face-to-face teaching provides a more immersive learning environment, as students are able to interact and collaborate directly with their instructors and peers. However, it was difficult to implement if the staff were unable to meet in person due to travel restrictions or social distancing measures. Additionally, face-to-face teaching is more time-consuming, as it requires instructors and students to be physically present during the course. Overall, both online and face-to-face teaching have advantages and limitations when used in a design studio course. Ultimately, it is important for instructors to consider their specific situations and available resources when deciding which teaching method is best for their course.

In this study, blended teaching in design studio courses offered many benefits to the students and staff. By combining traditional face-to-face instruction with online components, blended teaching allows students to benefit from both. For example, students can access online resources, such as lectures and demonstrations, from anywhere, allowing them to review as needed to gain a thorough understanding of the concepts presented. Additionally, blended teaching can provide students with the opportunity to work at their own pace and on their own schedule, increasing the likelihood of successful course completion. The benefits of blended teaching also extend to the staff. Additionally, the use of online components allows staff to have more control over the pacing of the course and to be able to cover more material in the allotted time. Overall, blended teaching offers many benefits to both the students and staff. Students are able to access online resources, work at their own pace, and access up-to-date information and materials.

Previously, the new technologies were limited to specific sectors, but nowadays, their low cost makes them affordable to promote learning outcomes in education. This research highlights the need to apply changes in online learning by developing the design program’s pedagogy. This incorporation of new technology and digital media into design pedagogy has the potential to support online design studio learning environments. ^21^ Furthermore, blended learning is not a novel learning system for universities that need a different mode of design pedagogy. Although students use the new technology, they still need more support and guidance, especially first-year students, to improve the quality and outcome of their design projects. ^20^^,^^21^ Several studies have been conducted on the impact of COVID-19 on various aspects of the design industry, including interior design. Some of the key aims of these studies have been to understand the challenges faced by designers during the pandemic and identify strategies for adapting to the new realities of remote work and social distancing. For example, Ref. 22 a study published in 2020 explored the challenges faced by design educators in transitioning to online teaching during a pandemic. This study identified several key challenges, including technological barriers, difficulties in adapting to new teaching methods, and reduced opportunities for hands-on learning. ^23^ A recent study examined the impact of the pandemic on interior design students’ learning experience. The study found that students faced significant challenges in adapting to online learning but also identified several strategies that could be used to enhance their learning experiences in the future. Overall, these studies highlight the importance of adapting to new technologies and teaching methods to continue delivering high-quality interior design education during the pandemic. By adopting a blended approach to studio practice and combining both online and in-person learning opportunities, educators and students can continue to develop their skills and expertise despite the challenges posed by COVID-19.

The COVID-19 pandemic has led to significant changes in the way businesses operate; interior design studios are no exception. As social distancing and remote work protocols have become the new norms, traditional studio-based practices have had to adapt to accommodate the restrictions imposed by the pandemic. A blended model of interior design studio practices has emerged as a way to balance the demands of client work with the need for safety and flexibility. This model combines in-person and remote work using technologies, such as video conferencing, file sharing, and collaborative software, to maintain team communication and project management. By allowing for both remote and in-person work, designers can also schedule client consultations in a way that balances the need for safety with the need for face-to-face interaction. A recent study showed that this blended model has proven to be effective, with interior design studios successfully completing projects on time and within budget, while maintaining the safety of both employees and clients. Compared to previous studies, this approach to interior design studio practice has been shown to be resilient and adapted to the current pandemic landscape. As the circumstances continue to evolve, it is likely that the blended model will remain a viable option for interior designers looking to navigate the post-pandemic landscape. ^21^

Recent studies have explored the efficacy of a blended model in interior design studio practices in light of the COVID-19 pandemic. This model combines both in-person and online instruction and collaboration, allowing for social distancing and safer working conditions, while maintaining the benefits of traditional studio practice. ^17^ One study found that students successfully engaged in design collaboration and critique through online platforms, suggesting that virtual communication tools could become an integral part of the design process even after the pandemic. However, the same study also highlighted the challenges of monitoring student progress in a virtual environment and maintaining a studio culture of mentorship and collaboration. ^15^ Another study examined the use of augmented reality and virtual reality tools in the design process, which could enhance the collaborative experience of students and create a more immersive design experience. ^18^^,^^22^ While technology is still developing, it holds promise for supporting the blended model and even expanding the possibilities of interior design education.

The results of this study showed that blended design studios achieved pedagogical results as students developed their knowledge and skills, especially in computer software. Students in various design studio courses now more widely appreciate blended learning in design studios due to in-depth study, literature reviews, and data discoveries. The benefits of combining in-person and online interactions create a much more inclusive learning environment than one-on-one instruction, as noted by Sopher. ^6^ The data collection shows that Ahlia University’s design studio’s use of blended learning has a high success rate, and most participants believe it is flexible. The comprehensive criteria of accessibility, preference, practicability, and student needs are all things blended learning attempts to address. The study concentrated on the many levels of design studio education’s legitimacy, influencing the next generation of architects and designers through technical and online education.

The alternative to blended learning is changing how architecture and interior design are taught, and this will likely be the following significant change in higher education that must be prepared. For students and professors, the ideal balancing act of physical and virtual learning opens up new ideas and opportunities to investigate the philosophy of distant learning and preserve practicality by attending physical design studio workshops. As previously mentioned, Ahlia University performed virtual learning using a variety of communication channels, allowing faculty members and students to experiment with various forms of engagement, including emails, text messages, audio calls, and video calls. The global COVID-19 pandemic necessitates the search for potential replacements where the educational framework of design studio classes is not jeopardized by a lack of communication, method learning, and practical experience. As a result, blended learning is crucial at this point in the pandemic to be acknowledged as a viable alternative to our everyday physical learning, which is impractical given the state of the world’s health. When it comes to meeting the requirements of traditional design studio sessions, blended learning is the ideal fit for the new reality of design education. Additionally, the results demonstrated that throughout the COVID-19 epidemic, all partners contributed to ensuring the caliber of online design studio instruction.

## Limitations

While this article presents a compelling argument for this blended model, it is important to note the limitations of the study. First, the study may not be generalizable to all interior design programs because it is based on the experience of one course. Different programs may have different teaching approaches and face different challenges. However, this study did not address the long-term effectiveness of the blended model. While it may have been effective in the short term because of the pandemic, it is unclear whether this model will continue to be effective in the future or if it will need to be modified as circumstances change. Overall, while the proposed blended model of interior design studio practice is promising, further research is required to fully understand its effectiveness and feasibility in various contexts.

## Conclusion

The results of this study showed that blended design studios achieved pedagogical results as students developed their knowledge and skills, especially in computer software. The blended model of interior design studio practice has proven to be a successful approach for adapting to the challenges brought about by COVID-19. By combining virtual and in-person experiences, students and professionals can continue their work in a safe and efficient manner. The virtual aspect of the blended model allows for greater flexibility and accessibility, whereas the in-person component provides hands-on learning and collaboration opportunities. This hybrid approach also encourages the use of technology and problem-solving skills, which are essential in the current industrial landscape. The blended model is a valuable tool for interior design education and practice, and has the potential to continue beyond the pandemic. As we navigate this new normal, it is important to continue exploring innovative solutions that prioritize safety while maintaining the quality and effectiveness of our work.

## Data Availability

A BLENDED MODEL OF INTERIOR DESIGN STUDIO PRACTICE DUE TO COVID-19 – Underlying Data. https://doi.org/10.6084/m9.figshare.21543417.v2. ^24^ This project contains the following underlying data:
- Underlying data.xlsx (responses to the survey) - Interview – Underlying data.xlsx (interview responses) Figshare: Blended Learning - Questionaire.pdf. https://doi.org/10.6084/m9.figshare.21940130.v1. ^25^ Data are available under the terms of the Creative Commons Attribution 4.0 International license (CC-BY 4.0).
