# Supplementary material for: A blended model of interior design studio practice due to COVID-19
Source: F1000Res. 2023 Oct 31;12:411. Originally published 2023 Apr 17. [Version 3] doi: 10.12688/f1000research.128478.3 (PMC10390791; doi:10.12688/f1000research.128478.3)
Supplement: Peer review history

# Reviewer report: Reviewer response for version 2

**DOI:** 10.5256/f1000research.147994.r184325

**Published:** 2023-07-31

**Authors:** Hermie E Delport

**Affiliations:** 1 University of Cape Town, Rondebosch, Western Cape, South Africa

I enjoyed the article and agree with you that the way forward is blended learning. It is an interesting study and it is thorough but the paper needs to be reviewed again and properly edited.

 The method has a biased outlook in the way that it is written. “After completing the INTD 212, INTD 216, INTD 311, and INTD 404 studios in mid-March 2022, a short Qualtrics poll was done to assess the difficulties of e-learning and offer potential consequences.” To say that you assess for the DIFFICULTIES of e-learning is showing anticipation that you will find difficulties and that might have influenced the way you did the observation/ designed the survey. One would rather say “to assess the implementation of e-learning”. You put it better put here “this study’s primary goal is to evaluate the effectiveness and standard of blended learning in design studio procedures as a result of COVID-19”.

 In general: Make sure that everything is in the past tense. E.g. you would need to change sentences such as “However, to finish the academic year without squandering the current semester, colleges worldwide are switching to online and e-learning methods.” Some sentences are not clear in what it means, such as “McGee and Reis ^18^ estimates that 30% and 79% of blended learning lessons are provided online.” There are strange sentences such as “None of the patients withdrew or dropped out of the study”. You are using “however” too much.

 I cannot find evidence that Schon wrote about the virtual design studio, as cited under the heading “Designing blended learning courses”.

 There are statements made and conclusion reached under the methodological section headings “Data analysis” and “Integration of Moodle and MS Teams”, such as “They discovered its effectiveness in the delivery of the courses.”

 The results section is interesting but not well organised and there are sentences and information that repeat.

Is the work clearly and accurately presented and does it cite the current literature?

Partly

If applicable, is the statistical analysis and its interpretation appropriate?

I cannot comment. A qualified statistician is required.

Are all the source data underlying the results available to ensure full reproducibility?

Partly

Is the study design appropriate and is the work technically sound?

No

Are the conclusions drawn adequately supported by the results?

Partly

Are sufficient details of methods and analysis provided to allow replication by others?

Partly

Reviewer Expertise:

Architectural education, studio and design education, design-build, AI in architecture, community engagement.

I confirm that I have read this submission and believe that I have an appropriate level of expertise to confirm that it is of an acceptable scientific standard, however I have significant reservations, as outlined above.

# Reviewer report: Reviewer response for version 2

**DOI:** 10.5256/f1000research.147994.r184318

**Published:** 2023-07-10

**Authors:** Martin William Andrews

**Affiliations:** 1 University of Portsmouth, Portsmouth, England, UK

This is an interesting article; thank you for submitting.

 I only have a few comments to make, in addition to the comments of my Peer Review colleague:

1. I would like you to include more, specific problems, that you think exist with the use of VLE when teaching design students to students in Higher Education.  Stating, "challenges" in the abstract, and, "unheard-of difficulties" in the Introduction, is too narrow and should be expanded upon in more detail. The Problem Statement starts to comment on these "challenges" but the paragraph could be expanded further to include actual scenario-based examples. 
2. There are several typographical errors throughout the text; but I am sure that these will be corrected at the final review/pre-publishing stage.
3. Particularly in the early part of the article, the word, "However" is used too frequently.

Is the work clearly and accurately presented and does it cite the current literature?

Yes

If applicable, is the statistical analysis and its interpretation appropriate?

I cannot comment. A qualified statistician is required.

Are all the source data underlying the results available to ensure full reproducibility?

Yes

Is the study design appropriate and is the work technically sound?

Yes

Are the conclusions drawn adequately supported by the results?

Yes

Are sufficient details of methods and analysis provided to allow replication by others?

Yes

Reviewer Expertise:

Architecture Education and Pedagogy/Design Education/Professional Development/Tutor Training

I confirm that I have read this submission and believe that I have an appropriate level of expertise to confirm that it is of an acceptable scientific standard, however I have significant reservations, as outlined above.

# Reviewer report: Reviewer response for version 2

**DOI:** 10.5256/f1000research.147994.r179991

**Published:** 2023-06-30

**Authors:** Noor Dheyaa Azeez

**Affiliations:** 1 Electronic Management Techniques, Northern Technical University, Mosul, Nineveh Governorate, Iraq

There are no additional comments on the revised version of the article.

Is the work clearly and accurately presented and does it cite the current literature?

Yes

If applicable, is the statistical analysis and its interpretation appropriate?

Partly

Are all the source data underlying the results available to ensure full reproducibility?

Yes

Is the study design appropriate and is the work technically sound?

Yes

Are the conclusions drawn adequately supported by the results?

Partly

Are sufficient details of methods and analysis provided to allow replication by others?

Yes

Reviewer Expertise:

Information technology, information systems, e-learning, mobile government, e-commerce, decision-making systems.

I confirm that I have read this submission and believe that I have an appropriate level of expertise to confirm that it is of an acceptable scientific standard.

# Reviewer report: Reviewer response for version 1

**DOI:** 10.5256/f1000research.141071.r170043

**Published:** 2023-04-25

**Authors:** Noor Dheyaa Azeez

**Affiliations:** 1 Electronic Management Techniques, Northern Technical University, Mosul, Nineveh Governorate, Iraq

The topic of study is vital and important.

 However, there are a set of observations that the researcher must follow:

1. The article did not mention the previous studies from which it was based. It is preferable to make a table that includes the previous studies, including the name of the study, the year, the title of the study, and the aim of the study. Then discuss these studies.
2. In the discussion section, the researcher did not compare the results of the article with the previous studies.
3. At the end of his article, the researcher did not provide a section for conclusion.
4. The researcher did not present the limitations of the article.
5. The article needs to enhance the statistical analysis method through the use of more advanced analytics.

Is the work clearly and accurately presented and does it cite the current literature?

Yes

If applicable, is the statistical analysis and its interpretation appropriate?

Partly

Are all the source data underlying the results available to ensure full reproducibility?

Yes

Is the study design appropriate and is the work technically sound?

Yes

Are the conclusions drawn adequately supported by the results?

Partly

Are sufficient details of methods and analysis provided to allow replication by others?

Yes

Reviewer Expertise:

Information technology, information systems, e-learning, mobile government, e-commerce, decision-making systems.

I confirm that I have read this submission and believe that I have an appropriate level of expertise to confirm that it is of an acceptable scientific standard, however I have significant reservations, as outlined above.

# Response

**Published:** 2023-05-08

**Authors:** Imad Assali

**Affiliations:** Ahlia University, Bahrain

- The article did not mention the previous studies from which it was based. It is preferable to make a table that includes the previous studies, including the name of the study, the year, the title of the study, and the aim of the study. Then discuss these studies.

**Answer: **Thank you for the comments. Based on the comments, the details have been added in the revised article.

^20, 21^Several studies have been conducted on the impact of COVID-19 on various aspects of the design industry, including interior design. Some of the key aims of these studies have been to understand the challenges faced by designers during the pandemic and identify strategies for adapting to the new realities of remote work and social distancing. For example, ^24^a study published in 2020 explored the challenges faced by design educators in transitioning to online teaching during a pandemic. This study identified several key challenges, including technological barriers, difficulties in adapting to new teaching methods, and reduced opportunities for hands-on learning. ^25^A recent study examined the impact of the pandemic on interior design students' learning experience. The study found that students faced significant challenges in adapting to online learning but also identified several strategies that could be used to enhance their learning experiences in the future. Overall, these studies highlight the importance of adapting to new technologies and teaching methods to continue delivering high-quality interior design education during the pandemic. By adopting a blended approach to studio practice and combining both online and in-person learning opportunities, educators and students can continue to develop their skills and expertise despite the challenges posed by COVID-19.

 References: DeCoito I, Estaiteyeh M. Transitioning to Online Teaching During the COVID-19 Pandemic: an Exploration of STEM Teachers' Views, Successes, and Challenges. J Sci Educ Technol. 2022;31(3):340-356.

 Ulusoy, B. (2023). Effects of COVID-19 Pandemic on Students’ Written Outcomes: An Interior Architecture Research/Theory Module Case Study in the UK. *Education Sciences*, *13*(1), 71.

  

- In the discussion section, the researcher did not compare the results of the article with the previous studies. 

**Answer: **Thank you for the comments. Based on the suggestions given, the results of this study were compared with previous studies.

 The COVID-19 pandemic has led to significant changes in the way businesses operate; interior design studios are no exception. As social distancing and remote work protocols have become the new norms, traditional studio-based practices have had to adapt to accommodate the restrictions imposed by the pandemic. A blended model of interior design studio practices has emerged as a way to balance the demands of client work with the need for safety and flexibility. This model combines in-person and remote work using technologies, such as video conferencing, file sharing, and collaborative software, to maintain team communication and project management. By allowing for both remote and in-person work, designers can also schedule client consultations in a way that balances the need for safety with the need for face-to-face interaction. A recent study showed that this blended model has proven to be effective, with interior design studios successfully completing projects on time and within budget, while maintaining the safety of both employees and clients. Compared to previous studies, this approach to interior design studio practice has been shown to be resilient and adapted to the current pandemic landscape. As the circumstances continue to evolve, it is likely that the blended model will remain a viable option for interior designers looking to navigate the post-pandemic landscape.

^21^Recent studies have explored the efficacy of a blended model in interior design studio practices in light of the COVID-19 pandemic. This model combines both in-person and online instruction and collaboration, allowing for social distancing and safer working conditions, while maintaining the benefits of traditional studio practice. ^17^One study found that students successfully engaged in design collaboration and critique through online platforms, suggesting that virtual communication tools could become an integral part of the design process even after the pandemic. However, the same study also highlighted the challenges of monitoring student progress in a virtual environment and maintaining a studio culture of mentorship and collaboration. ^15^Another study examined the use of augmented reality and virtual reality tools in the design process, which could enhance the collaborative experience of students and create a more immersive design experience. ^18, 24^While technology is still developing, it holds promise for supporting the blended model and even expanding the possibilities of interior design education.

  

- At the end of his article, the researcher did not provide a section for conclusion.

**Answer: **Thank you for the comments. Based on the comments, conclusion section is added.

 The results of this study showed that blended design studios achieved pedagogical results as students developed their knowledge and skills, especially in computer software. The blended model of interior design studio practice has proven to be a successful approach for adapting to the challenges brought about by COVID-19. By combining virtual and in-person experiences, students and professionals can continue their work in a safe and efficient manner. The virtual aspect of the blended model allows for greater flexibility and accessibility, whereas the in-person component provides hands-on learning and collaboration opportunities. This hybrid approach also encourages the use of technology and problem-solving skills, which are essential in the current industrial landscape. The blended model is a valuable tool for interior design education and practice, and has the potential to continue beyond the pandemic. As we navigate this new normal, it is important to continue exploring innovative solutions that prioritize safety while maintaining the quality and effectiveness of our work.

  

- The researcher did not present the limitations of the article.

**Answer: **Thank you for the comments. Based on the comments, limitations of the study are added.

 While this article presents a compelling argument for this blended model, it is important to note the limitations of the study. First, the study may not be generalizable to all interior design programs because it is based on the experience of one course. Different programs may have different teaching approaches and face different challenges. However, this study did not address the long-term effectiveness of the blended model. While it may have been effective in the short term because of the pandemic, it is unclear whether this model will continue to be effective in the future or if it will need to be modified as circumstances change. Overall, while the proposed blended model of interior design studio practice is promising, further research is required to fully understand its effectiveness and feasibility in various contexts.

  

- The article needs to enhance the statistical analysis method through the use of more advanced analytics

**Answer: **Than you for the comments. Based on the comments, the details have been updated in the revised article.

 Enhancing statistical analysis methods through advanced analytics is essential to provide designers with the data-driven insights they need to create more valuable and customer-centric interior design solutions. It is a powerful tool that provides the ability to easily perform intricate analyses. Moreover, it includes a comprehensive range of statistical procedures that are useful for analyzing complex datasets. With the onset of the COVID-19 pandemic, interior design studio practices have been significantly impacted. The adoption of a blended model can help in mitigating the effects by enabling online collaboration. Using SPSS version 27.0.1.0 to analyzing data from the blended model can enable practitioners to derive meaningful insights from the collected data.
